# Epidemiology of Nontuberculous Mycobacteria Infection in Children and Young People With Cystic Fibrosis: Analysis of UK Cystic Fibrosis Registry

**DOI:** 10.1093/cid/ciy531

**Published:** 2018-07-05

**Authors:** Aaron I Gardner, Elliot McClenaghan, Gemma Saint, Paul S McNamara, Malcolm Brodlie, Matthew F Thomas

**Affiliations:** 1 Institute of Cellular Medicine, Faculty of Medical Sciences, Newcastle University, Newcastle upon Tyne; 2 Department of Child Health, University of Liverpool; 3 Alder Hey Children’s National Health Service Foundation Trust; 4 Department of Paediatric Respiratory Medicine, Great North Children’s Hospital, Newcastle upon Tyne Hospitals National Health Service Foundation Trust, United Kingdom

**Keywords:** cystic fibrosis, nontuberculous mycobacteria, children, NTM, epidemiology

## Abstract

**Background:**

Infection with nontuberculous mycobacteria (NTM) is of growing clinical concern in people with cystic fibrosis (CF). The epidemiology of infection in children and young people remains poorly understood. Our goal was to investigate the epidemiology of NTM infection in the pediatric age group using data from the UK CF Registry.

**Methods:**

Data from 2010–2015 for individuals aged <16 years (23200 observations from 5333 unique individuals) were obtained. Univariate analysis of unique individuals comparing all key clinical factors and health outcomes to NTM status was performed. The significant factors that were identified were used to generate a multivariate logistic regression model that, following step-wise removal, generated a final parsimonious model.

**Results:**

The prevalence of individuals with a NTM-positive respiratory culture increased every year from 2010 (45 [1.3%]) to 2015 (156 [3.8%]). Allergic bronchopulmonary aspergillosis (odds ratio [OR], 2.66; *P* = 5.0 × 10^−8^), age (OR, 1.08; *P* = 3.4 × 10^−10^), and intermittent *Pseudomonas aeruginosa* infection (OR, 1.51; *P* = .004) were significantly associated with NTM infection.

**Conclusions:**

NTM infection is of increasing prevalence in the UK pediatric CF population. This study highlights the urgent need for work to establish effective treatment and prevention strategies for NTM infection in young people with CF.

## Teaser

The prevalence of nontuberculous mycobacteria (NTM) infection is increasing in children and young people with cystic fibrosis. There is an urgent need to establish the most effective strategies to prevent and treat NTM infection in the pediatric age group.

More than 10000 individuals have cystic fibrosis (CF) in the United Kingdom, making it the most common inherited life-limiting condition [1]. More than 4000 are aged <16 years [1]. The CF Trust maintains a detailed UK patient registry that records anonymized clinical data and health outcomes [2]. The registry is updated annually and captures information that covers more than 90% of people with CF nationally [1, 2]. Despite the development of specialist care and incremental improvements in survival, lung disease still accounts for the substantial majority of morbidity and premature mortality in people with CF [3]. Susceptibility to respiratory infection with particular microbes is a key component of the pathology of CF lung disease, and approaches that target the treatment or prevention of infection are a mainstay of clinical management [3].

Over the last 5 years, infection with nontuberculous mycobacteria (NTM) has become a subject of increasing clinical concern in people with CF [4]. NTM are environmental organisms found commonly in soil and water. Two groupings are most frequently isolated from people with CF, *Mycobacterium abscessus* complex and *Mycobacterium avium* complex. Infection with *M. abscessus* specifically has been found to be associated with increased decline in lung function [5, 6]. Treatment of airway infection with NTM requires prolonged courses of multiple antibiotics, often for more than 12 months, and is associated with a significant treatment burden and frequent adverse effects [4, 7]. Furthermore, *M. abscessus* infection is regarded as a relative contraindication to lung transplantation in many centers [8]. There is also emerging evidence to suggest transmission of *M. abscessus* between individuals with CF [9, 10].

There are varying estimates of the prevalence of NTM isolation in respiratory samples from people with CF that range from 3.7% in parts of Europe to 14% in the United States [11, 12]. Several reports have suggested an increasing prevalence over the last decade above that accounted for by improved screening and detection techniques [5, 13–16]. A previous European registry study of a combined adult and pediatric CF population identified age; allergic bronchopulmonary aspergillosis (ABPA); *Stenotrophomonas maltophilia* infection; and use of bronchodilators, inhaled antibiotics, or rhDNase to be associated with NTM infection [17]. Specific knowledge of the epidemiology of NTM infection in children and young people with CF is particularly limited at present. Paradoxically it is in the pediatric age group where interventions and strategies to treat or prevent infection with NTM are likely to yield the greatest clinical benefits.

We analyzed data from the UK CF Registry to investigate the epidemiology of NTM infection in individuals aged <16 years between 2010 and 2015. This included trends in the prevalence of NTM infection, individual species isolated, demographic and clinical “risk factors” in individuals isolating NTM, along with longitudinal analyses. These data and analyses will inform future clinical research relating to NTM infection in children and young people with CF.

## METHODS

### Clinical Data and Research Ethics

Annual review data submitted to the UK CF Registry database between 2010 and 2015 for patients aged <16 years (23200 observations from 5333 unique individuals) were obtained. Supplementary Table 1 lists the variables obtained. Data lacking a unique identifying code were excluded from the analysis (n = 38).

Explicit written consent is obtained from individuals or their parents or guardians for their inclusion in the UK CF Registry. The registry is compliant with UK data protection legislation and subject to continued Research Ethics Committee approval. Data for this study were obtained by application to the CF Trust Research Registry Committee, which approved the application and released anonymized data in line with the existing registry ethics approvals.

### Data Analyses

The data were first cleaned and checked for duplicates. For each individual, NTM-positive status was defined using the specific registry field, which is returned if an individual has had a positive NTM culture in the preceding year. Intrinsically, this was not whether an individual met the criteria for NTM-related pulmonary disease (NTM-PD) but purely whether they had a positive culture in that 12-month period [18]. In the 2014 and 2015 registry censuses, this field was expanded to collect more detailed data about NTM status, including species, date of culture, and treatment data. Individuals were tracked through the time period using their unique registry ID.

All unique individuals were then pooled for analysis of epidemiological risk factors predictive of NTM status. These included demographics (age, sex, anthropometric measurements), *CFTR* genotype, lung function, other respiratory microbiological status (*P. aeruginosa, Staphylococcus aureus*, and *Burkholderia cepacia*; *S. aureus* and *P. aeruginosa* were subdivided into 3 states: negative; intermittent, defined as 1–2 isolations in the last year; or chronic, defined as 3 or more isolations in the last year), and comorbidities (CF-related diabetes or ABPA).

Following a standard approach, univariate nonparametric tests were used to assess predictive value. All significant predictors were then included in a multivariate logistic regression model. Step-wise removal of nonsignificant factors was then undertaken to generate the final parsimonious model presented. For variables with multiple categorical states included in the multivariate model, for example, *P. aeruginosa* status, an overall effect was estimated using the Wald test.

Next, the progress of patients’ NTM status was tracked over the entire time period to generate annual incidence, prevalence, and potential response to treatment estimates. For patients who remained NTM positive or NTM negative for the duration of the study period, only their latest time point was included for analysis. Those with multiple classifications, that is, those who developed or were cleared of NTM infection during the study period, were classified as NTM positive, and data from their latest NTM-positive time point were included for analysis.

Finally, descriptive data on NTM species and treatment were collated and explored for the 2014 and 2015 data extracts. All analyses were carried out using Microsoft Access 365, GraphPad Prism 6.05, and R 3.4.1 using the *nlme* package [19].

## RESULTS

**Figure 1.**
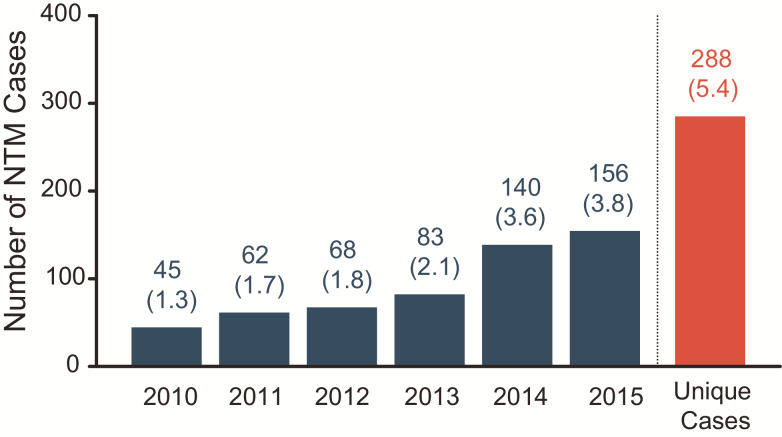
Nontuberculous mycobacteria (NTM) prevalence by year. The prevalence of NTM in cystic fibrosis patients increased each year, with a particularly large increase observed between 2013 and 2014. After merging of the data, the number of unique cases overall (288, 5.4%) was identified. Values in brackets show percentage of individuals positive for NTM per year.

The prevalence of individuals with a NTM-positive respiratory culture in the UK CF Registry aged <16 years increased every year from 2010 (45 [1.3%]) through 2015 (156 [3.8%]; Figure 1). An especially large increase in cases was observed between 2013 (83 [2.1%]) and 2014 (140 [3.6%]). Following the pooling of yearly cohorts, the number of overall unique NTM cases was identified (288 from 5333 [5.4%]).

Table 1 summarizes the differences between those children and young people who had an NTM-positive respiratory culture and those who did not. On average, children who isolated NTM were older (median 10 years vs 6 years, *P* = 2.2 × 10^–16^), were more likely to be positive for *P. aeruginosa* (45% vs 30%*, P* = .013), more likely to be positive for *B. cepacia* (2.4% vs 0.9%, *P* = .013), and more likely to have ABPA (17% vs 4.7%, *P* = 2.2 × 10^–16^).

**Table 1.** Summary of Merged Registry Data for 2010–2015 and Univariate Analysis of Risk Factors for Nontuberculous Mycobacteria–positive Cultures

	All		NTM		NTM Negative		
Variable	n (%)	Median (Quartiles)	n (%)	Median (Quartiles)	n (%)	Median (Quartiles)	*P* Value
Patients	5333 (100)	…	288 (5.4)	…	5045 (94.6)	…	…
Demographics							
Male	2717 (50.9)	…	138 (47.9)	…	2579 (51.1)	…	.554
Female	2615 (49)	…	150 (52.1)	…	2465 (48.9)	…	
Age, years	5333 (100)	6 (2–12)	288 (100)	10 (7–12)	5045 (100)	6 (2–12)	**2.2 × 10** ^**−16**^
Height percentile	5110 (95.8)	112.2 (79.6–142.6)	282 (97.9)	133.9 (117.7–145.9)	4828 (95.7)	110 (78.7–142)	.020
Weight percentile	5235 (98.2)	19.3 (10.9–34.9)	281 (97.6)	28.8 (21.5–37.6)	4954 (98.2)	18.6 (10.6–34.7)	.420
Body mass index percentile	3846 (72.1)	52.8 (28.2–76.1)	262 (91)	44.3 (22.1–70.48)	3224 (63.9)	53.48 (28.5–76.53)	.850
Genotype							
*F508del F508del*	2704 (50.7)	…	164 (56.9)	…	2540 (50.3)	…	.418
*F508del*/Other	1968 (36.9)	…	89 (30.9)	…	1879 (37.2.	…	.452
Other/Other	661 (12.4)	…	35 (12.2)	…	626 (12.4)	…	.469
Lung function							
FEV_1_	2541 (47.6)	1.64 (1.2–2.2)	64 (22.2)	1.5 (1.2–1.9)	2728 (54.1)	1.7 (1.2–2.2)	.991
FEV_1_% predicted	2271 (42.6)	85.9 (73.4–97.5)	76 (26.4)	81.3 (68.2–94.1)	2986 (59.2)	86.4 (74.3–97.8)	.286
Respiratory microbiology							
*Staphylococcus aureus*	1298 (24.3)	…	85 (29.5)	…	1213 (24)	…	…
Chronic	319 (6)	…	23 (8)	…	296 (5.9)	…	.093
Intermittent	979 (18.4)	…	62 (21.5)	…	917 (18.2)	…	.103
*Pseudomonas aeruginosa*	1640 (30.8)	…	130 (45.1)	…	1510 (29.9)	…	.013^a^
Chronic	505 (9.5)	…	45 (15.6)	…	460 (9.1)	…	**8.65 × 10** ^−**06**^
Intermittent	1135 (21.3)	…	85 (29.5)	…	1050 (20.8)	…	**1.93 × 10** ^**−05**^
*Burkholderia cepacia*	54 (1)	…	7 (2.4)	…	47 (0.9)	…	**.013**
*Burkholderia cenocepacia*	13 (0.2)	…	2 (0.7)	…	11 (0.2)	…	.111
*Burkholderia multivorans*	25 (0.5)	…	3 (1)	…	22 (0.4)	…	.143
Comorbidities							
Allergic bronchopulmonary aspergillosis	285 (5.3)	…	49 (17)	…	236 (4.7)	…	**2.2 × 10** ^**−16**^
_Cystic fibrosis–related diabetes_	153 (2.9)	…	11 (3.8)	…	142 (2.8)	…	.321

Summary statistics for merged data. Thirty-eight data points lacking a unique identifying code were removed from the analysis, leaving a final count of 5333 patients. Univariate analysis comparing each factor to NTM status was performed and *P* values presented. Significant factors (in bold) were included in the final multivariate analysis.

Abbreviations: FEV_1_, forced expiratory volume exhaled at the end of the first second of forced expiration; NTM, nontuberculous mycobacteria.

^a^By Wald test, χ^2^ = 8.6, *P* = .013. Tested prior to inclusion in multivariate model.

No gender difference was seen, nor was there a difference between *F508del* (c.1521 1523del) homozygotes or heterozygotes. Heterozygosity for the *W1282X* (c.3846G>A) mutation was statistically significantly associated with positive NTM status. However, given the scarcity of this (21 heterozygotes) and other rare mutations in the dataset, the chance of statistical error was deemed too great for further analysis.

While, on average, individuals who had a respiratory culture positive for NTM had a lower body mass index percentile (44.3 vs 53.4, *P* = .850) and lower forced expiratory volume in 1 second percentile (81.3 vs 86.4, *P* = .286), neither was statistically significantly different. No difference was seen between rates of *S. aureus*, *Burkholderia cenocepacia*, and *Burkholderia multivorans* infection between the 2 groups. There was no statistically significant difference in the incidence of CF-related diabetes between the NTM-positive and NTM-negative groups (3.8% vs 2.8%, *P* = .321).

**Figure 2.**
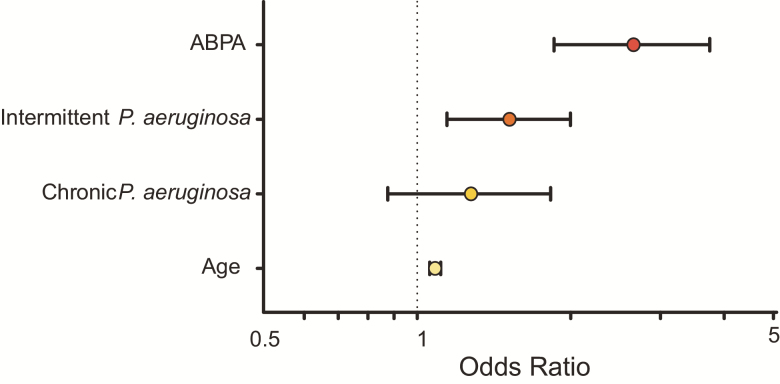
Odds ratios (ORs) for multivariate model. ORs and 95% confidence interval for allergic bronchopulmonary aspergillosis, chronic and intermittent *Pseudomonas aeruginosa*, and age from the multivariate model are displayed. Values are shown in Table 2. Abbreviation: ABPA, allergic bronchopulmonary aspergillosis.

Following univariate analysis and step-wise logistic regression, age, *P. aeruginosa*, *B. cepacia*, and ABPA status were incorporated into a multivariate model (Table 2, Figure 2). In the final model, increased age and ABPA were significantly associated with NTM status, with a larger effect observed for ABPA (age odds ratio [OR], 1.08; 95% confidence interval [CI], 1.06–1.11; *P* = 3.4 × 10^−10^ and ABPA OR, 2.66; 95% CI, 1.85–3.75; *P* = 5.0 × 10^−8^, respectively). Intermittent, but not chronic, *P. aeruginosa* colonization was also significantly associated (OR, 1.51; 95% CI, 1.14–1.99; *P* = .004). *B. cepacia* was not significantly associated and hence removed from the final parsimonious model.

**Table 2.** Odds Ratios and Significance for Multivariate Model

Variable	Odds Ratio	95% Confidence Interval	*P* Value
Allergic bronchopulmonary aspergillosis	2.656	1.854–3.748	5.0 × 10^-8^
_*Pseudomonas*_ *aeruginosa* intermittent	1.511	1.139–1.990	.004
*P. aeruginosa* chronic	1.275	0.875–1.825	.195
Age	1.084	1.057–1.111	3.4 × 10^-10^

Odds ratios, 95% confidence interval, and *P* values for allergic bronchopulmonary aspergillosis, chronic and intermittent *P. aeruginosa*, and age from the multivariate model are displayed.

**Figure 3.**
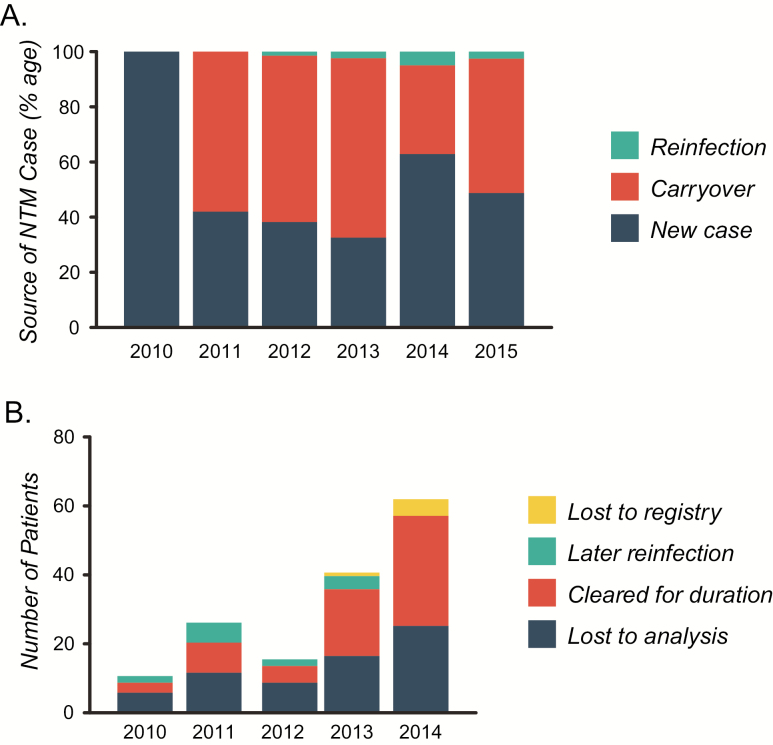
Nontuberculous mycobacteria (NTM) origin and patient outcomes. *A*, Individual patients were tracked through the registry, and the source of infection was graphed. Large increases in the total number and percentage of new cases were observed between 2013 and 2014. Values are shown in Table 3. *B*, Patient outcomes for those who did not carryover NTM-positive status into the following years analysis were also determined. A large increase in those who cleared NTM for the remainder of the assessment period was observed for 2013 and 2014. Values are shown in Table 4. “Lost to registry” refers to patients who should have remained available for analysis but lacked registry data for later years for unknown reasons. “Lost to analysis” refers to patients who became older than 16 years and so were no longer included in the analysis.

By following individual patients through the registry longitudinally, it was possible to determine the incidence of new cases as a proportion. Between 2011 and 2013, approximately 40% (approximately 26 cases per year) of NTM-positive individuals were new cases (Table 3, Figure 3A). As discussed above, a large increase in the overall number of NTM cases was observed between 2013 and 2014, which was accounted for by a large increase in the number of new cases arising that year (63% [88]).

**Table 3.** Nontuberculous Mycobacteria Origin

Year	New Cases (%)	Carryover (%)	Reinfection (%)	Total
2010	45 (100)	0 (0)	0 (0)	45
2011	26 (41.9)	36 (58.1)	0 (0)	62
2012	26 (38.2)	41 (60.3)	1 (1.5)	68
2013	27 (32.5)	54 (65.1)	2 (2.4)	83
2014	88 (62.9)	45 (32.1)	7 (5.0)	140
2015	76 (48.7)	76 (48.7)	4 (2.6)	156
Total (%)	288 (52.0)	252 (45.5)	14 (2.5)	554

Origin of nontuberculous mycobacteria cases per year, data visualized in Figure 3A.

**Table 4.** Nontuberculous Mycobacteria Patient Outcomes

Year	Lost to Analysis (%)	Cleared for Duration (%)	Later Reinfection (%)	Lost to Registry (%)	Total
2010	6 (13.3)	1 (2.2)	2 (4.4)	0 (0)	9
2011	12 (19.4)	3 (4.8)	6 (9.7)	0 (0)	21
2012	9 (13.2)	3 (4.4)	2 (2.9)	0 (0)	14
2013	17 (20.5)	16 (19.3)	4 (4.8)	1 (1.2)	38
2014	26 (18.6)	33 (23.6)	0 (0)	5 (3.6)	64
Total	70	56	14	6	

Nontuberculous mycobacteria patient outcomes per year, data visualized in Figure 3B.

We then assessed the outcomes for patients who did not carryover NTM-positive status into the following year’s analysis based on the registry data. Between 2010 and 2012, approximately 2 (approximately 4%) patients per year cleared NTM for the duration of the study (Table 4, Figure 3B). In 2013 and 2014, there was a marked increase in this number up to 16 (19.3%) and 33 patients (23.6%), respectively. However, it is likely that some of this effect is due to the endpoint of existing data.

**Figure 4.**
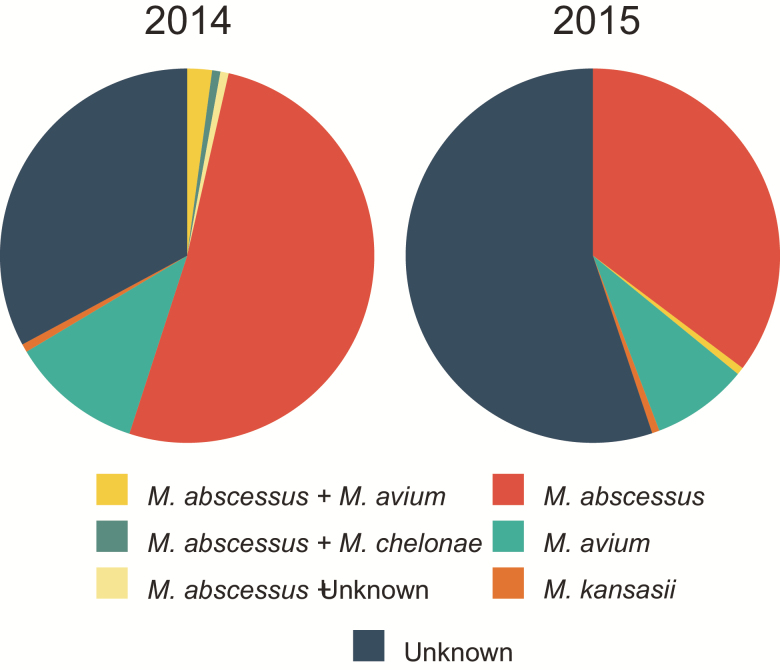
Specific strain information for 2014–2015. For 2014–2015, the registry introduced nontuberculous mycobacteria typing information. Some variation between years was observed, especially in relation to the number of unknown cases. *Mycobacterium abscessus* and *Mycobacterium* avium accounted for the majority of cases for each year. Values are shown in Supplementary Table 2.

From 2014 the registry began including NTM typing information from patient isolates and treatment information where available. For both 2014 and 2015, *M. abscessus* (72 [51.4%] and 55 [35.3%], respectively) and *M. avium* (16 [11.4%] and 13 [8.3%], respectively) accounted for the majority of known infections (Figure 4, Supplementary Table 2). A large proportion of samples lacked specific typing information in 2014 (46 [32.9%]), which increased in 2015 (86 [55.1%]).

A wide variety of therapies were used in the treatment of NTM. Descriptive data are presented in Supplementary Table 3 on the different antimicrobials used. Treatments broadly followed accepted guidelines, with amikacin preferentially used for the treatment of *M. abscessus* over *M. avium* [4, 7]. In a small number of cases, ethambutol and rifampicin are recorded as having been used to treat *M. abscessus*, which is not in keeping with guidance and is likely to have been ineffective.

## DISCUSSION

We present data from the nationally representative UK CF Registry that show an increase in children and young people isolating NTM in respiratory cultures between 2010 and 2015. Over a 5-year period, there was a 3-fold increase in the prevalence of NTM-positive cultures, with approximately 5% of those aged <16 years included in the registry being NTM positive at some point during the study period. *M. abscessus* complex and *M. avium* complex accounted for the overwhelming majority of isolates. Similar to other cohorts, age and ABPA status appeared to be significant predictors of NTM culture positivity, in addition to *P. aeruginosa* status [17].

A major strength of this study is derived from the nationally representative longitudinal source data. The UK CF Registry is an internationally recognized resource, with more than 90% of individuals with CF in the United Kingdom included in the dataset. The methodology of data collection has remained consistent over the time period we report, meaning that we can have confidence that reporting bias should not be a factor in these estimates. Second, this dataset allows investigation and analysis of the epidemiology of NTM infection specifically in individuals aged <16 years and represents the largest pediatric cohort analyzed in this regard. There are important biological differences as CF lung disease progresses through a spectrum from the early stages in children to advanced disease in adults. Furthermore, children and young people receive care in pediatric centers that may adopt models of care that are different from those used in adult centers.

Conversely, the limitations of our study stem from the fundamental structure, as we had no direct control over reporting, and are inherent to registry-based research. The registry does not record data on the frequency of sampling in patients or the sample types used. However, all UK centers delivering care are expected to adhere to the 2010 CF Trust guideline, *Laboratory Standards for Processing Microbiological Samples from People with Cystic Fibrosis*. Furthermore, the *Standards for the Clinical Care of Children and Adults with Cystic Fibrosis in the UK*, published in 2011, states that all patients should have a respiratory sample cultured for NTM at least annually. It is possible, however, that surveillance on only a yearly basis may lead to underestimation of the true prevalence. In some cases, there was no recording of the individual species of NTM in the registry.

It is also important to state that the presence of positive respiratory cultures for NTM is not synonymous with NTM-PD as defined by the American Thoracic Society/Infectious Diseases Society of America 2007 statement [18]. The diagnosis of NTM-PD requires multiple clinical criteria to be met and is beyond the scope of this study.

The question of whether the increase in prevalence of NTM represents a true rise in NTM-PD in UK children with CF or simply represents better detection is a vexatious one. Similar questions have arisen in virtually every epidemiological study of infection, and the data we present cannot fundamentally differentiate between these 2 possibilities. Methods to culture and detect NTM in respiratory samples have developed over recent years. For example, a new selective NTM growth medium has been demonstrated to increase sensitivity of the detection of NTM, but this is used in a minority of UK CF centers [14, 20, 21]. Over the same time period, reports have suggested possible transmission of NTM, specifically *M. abscessus* complex, between individuals with CF despite the presence of routine infection control measures in CF centers, which would provide a potential mechanism for the observed increasing prevalence [9, 10, 22, 23].

Within our cohort, we found associations with increasing age (OR, 1.08; *P* = 3.4 × 10^−10^), ABPA (OR, 2.66; *P* = 5.0 × 10^−8^), and *P. aeruginosa* status (intermittent OR, 1.51; *P* = .004) and risk of NTM-positive respiratory samples. These were consistent findings over the different annual cohorts and were also found in the pooled analysis of all unique individuals. Viviani et al reported similar associations with age and ABPA, but not *P. aeruginosa*, in multivariate analysis of European registry data from both children and adults with CF combined [17].

It is conceivable that all 3 identified risk factors in our study represent surrogate markers of increased lung destruction or damage that has been postulated to increase an individual’s vulnerability to NTM acquisition in individuals without CF [24, 25]. Advancing age is also associated with an inherent increase in duration of time at risk of exposure. There may also be ecological interactions within the lung microbiome that predispose to coacquisition of *P. aeruginosa*, *Aspergillus* species, and NTM. Furthermore, host immune function is likely to be relevant to these risk factors. In people with chronic obstructive pulmonary disease, the use of inhaled corticosteroids has been found to be a risk factor for NTM infection [26]. ABPA is usually treated with corticosteroids, and it is conceivable that there is a similar association in people with CF.

Our finding of a difference in the association between NTM status and intermittent and chronic *P. aeruginosa* status is counterintuitive. The distribution of the CIs of the chronic status (Figure 2), however, strongly favors an effect, and the overall class effect (combining both chronic and intermittent status) is significant. We suspect that this difference would likely disappear in larger populations. It is worth noting that the UK CF Registry classification of *P. aeruginosa* status is based on the number of positive respiratory samples during the last 12 months (negative; intermittent, defined as 1–2 isolations; or chronic, defined as 3 or greater isolations), irrespective of whether or not the individual is maintained on chronic antipseudomonal suppression therapy, for example, nebulized antibiotics.

Our results highlight that NTM infection appears to be increasing significantly in the UK pediatric CF population. An observation made anecdotally and expressed readily at CF clinician meetings over the last 5 years. These data make a compelling argument for urgent investment in research of NTM infection in children and young people. The advances we have seen in clinical outcomes in CF have largely come from innovations and improvements in pediatric CF care. Similarly, by addressing NTM infection in childhood, the greatest potential gains can be made. Our data lead us to conclude that 2 areas in particular need urgent focus and collaboration. First, precise, detailed epidemiological data on NTM infection in childhood need to be obtained so that strategies to prevent acquisition can be developed. Second, urgent randomized trials of antimicrobial therapies directed against *M. abscessus* complex and *M. avium* complex in children and young people are required.

## Supplementary Data

Supplementary materials are available at *Clinical Infectious Diseases* online. Consisting of data provided by the authors to benefit the reader, the posted materials are not copyedited and are the sole responsibility of the authors, so questions or comments should be addressed to the corresponding author.

Supplementary Material

## Notes

***Acknowledgments.*** We are grateful to the Cystic Fibrosis Trust UK Research Registry Committee for approving and facilitating our access to the data analyzed in this research.

***Financial support.*** This work was supported by the Medical Research Council (UK) (grant MR/M008797/1 Clinician Scientist Fellowship to M.B.) and the National Institute for Health Research (NIHR) Newcastle Biomedical Research Centre based at Newcastle Hospitals National Health Service Foundation Trust and Newcastle University. The views expressed are those of the author(s) and not necessarily those of the NHS, the NIHR, or the Department of Health.

***Potential conflicts of interest.*** M.B., unrelated to this work, received investigator-led research grants from Pfizer and Roche Diagnostics; honoraria for speaking at educational meetings paid to Newcastle University from Novartis, TEVA, and Roche Diagnostics; and travel and accommodation for an educational meeting from Boehringer Ingelheim. M.F.T., unrelated to this work, received an investigator-led research grant from Pfizer. All remaining authors: No reported conflicts of interest. All authors have submitted the ICMJE Form for Disclosure of Potential Conflicts of Interest. Conflicts that the editors consider relevant to the content of the manuscript have been disclosed.
