# Erratum to: Epidemiology of Nontuberculous Mycobacteria Infection in Children and Young People With Cystic Fibrosis: Analysis of UK Cystic Fibrosis Registry

**DOI:** 10.1093/cid/ciaa448

**Published:** 2021-01-04

An error appeared in the 1 March 2019 issue of the journal [Gardner AI, McGlenaghan E, Saint G, et al. Epidemiology of Nontuberculous Mycobacteria Infection in Children and Young People With Cystic Fibrosis: Analysis of UK Cystic Fibrosis Registry. Clin Infect Dis 2019; 68(5):731–737. https://doi.org/10.1093/cid/ciy531].

Data that authors were provided for creating this article only included ‘people requiring treatment for nontuberculous mycobacteria (NTM)’ rather than ‘all people who had isolated NTM’, which likely caused an under ascertainment of total numbers in the analysis. A more comprehensive dataset has been reanalyzed (see below).

The data were reanalyzed on the basis of all those with a positive NTM event over the last year, regardless of whether they received treatment or not. Figure 1 shows the number of cases each year from 2010 to 2015. The cases in the original analysis are displayed in dark blue with red indicating the results of the reanalysis. In the reanalysis, there were most additional NTM cases identified from 2010 to 2013 (which fits with the database issues identified for those years by the registry team). This smoothed out somewhat the previously identified large increase from 2013 to 2014. The overall effect is a flattening of the trend slightly, but there was still a substantial increase in cases observed between 2010 to 2015.

**Figure 1.**
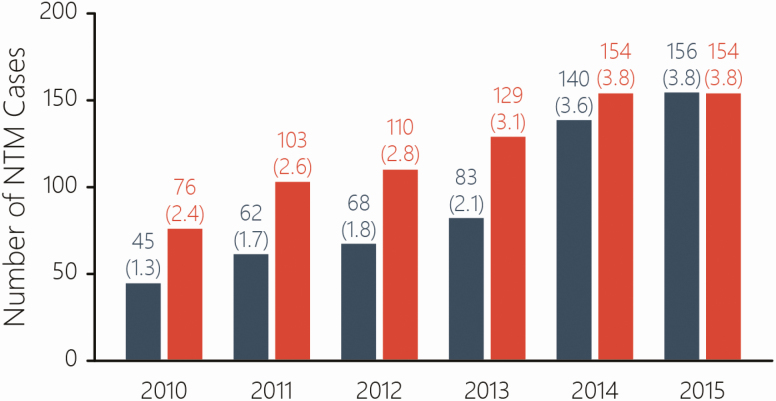
**Number of NTM cases 2010–2015.** Shown in dark blue are the original counts, with those in red showing the new analysis. While the actual counts from 2010–2013 have increased, the overall trend is the same.

The second part of the paper was a multivariate analysis of factors associated with NTM cases. This was based on factors that were present in all datasets from 2010–2015 and is shown in Figure 2.

**Figure 2.**
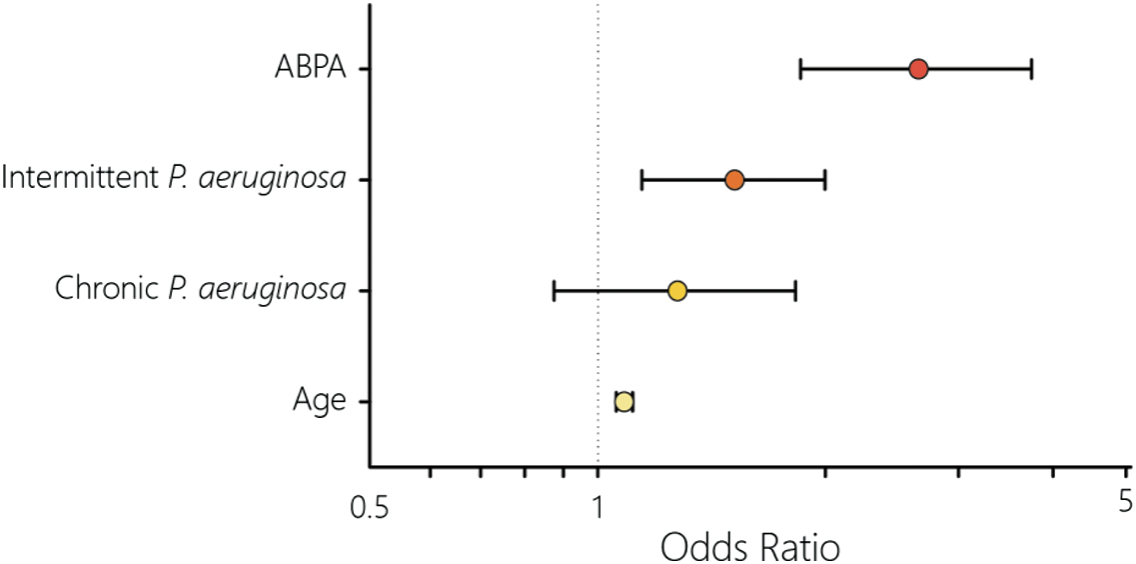
**Original multivariate analysis of factors associated with positive nontuberculous mycobacteria (NTM) status.** This identified four factors that were positively associated with NTM status: allergic bronchopulmonary aspergillosis (ABPA), *Pseudomonas aeruginosa* colonisation (intermittent but not chronic, effect remained when considering both together) and age.

The multivariate analysis was repeated, including the additional cases identified in the more comprehensive dataset. Figure 3 shows the results of the repeat analysis. Allergic bronchopulmonary aspergillosus (ABPA), being the strongest marker, and age remained relatively unchanged. *Pseudomonas aeruginosa* status swapped around with chronic rather than intermittent colonisation now being statistically significantly associated with NTM status. In both analyses, a positive association was observed when both chronic and intermittent *P. aeruginosa* infection are grouped together. Body mass index centile had a small but statistically significant positive association with NTM status in the repeat analysis. Both chronic and intermittent *Staphylococcus aureus* status were negatively associated with NTM status in the reanalysis.

**Figure 3.**
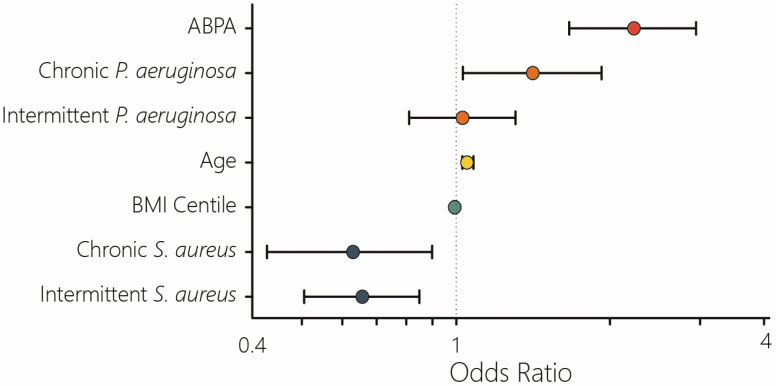
**Repeat multivariate analysis of factors associated with positive nontuberculous mycobacteria (NTM) status.** With the additional NTM cases identified the same analysis was performed again. Both age and ABPA status remained positively associated. However, while *Pseudomonas aeruginosa* colonization remained a positive factor the individual factors switched (chronic but not intermittent, effect remained when considering both together). Body mass index (BMI) centile was very slightly, but statistically significantly associated with NTM status. *Staphylococcus aureus* status (chronic and intermittent) were now negatively associated.

Overall, the authors feel that the primary conclusion and message from the paper, Re: Increased Children and Young People With CF Isolating NTM Between 2010 and 2015, is unchanged. However, in the reanalysis of the more comprehensive dataset, we have identified differences in the actual numbers and in some of the factors associated with NTM status in the multivariate analysis.

The authors apologize for the error.

