# Supplementary material for: Epidemiology of Nontuberculous Mycobacteria Infection in Children and Young People With Cystic Fibrosis: Analysis of UK Cystic Fibrosis Registry
Source: Clin Infect Dis. 2018 Jul 5;68(5):731–7. doi: 10.1093/cid/ciy531 (PMC6376093; doi:10.1093/cid/ciy531)
Supplement: Supplementary Material [file ciy531_suppl_supplementary_material.docx]

## SUPPLEMENTARY MATERIAL

## Epidemiology of nontuberculous mycobacteria infection in children and young people with cystic fibrosis: analysis of UK Cystic Fibrosis Registry

Aaron I. Gardner, Elliot McClenaghan, Gemma Saint, Paul S. McNamara, Malcolm Brodlie and Matthew F. Thomas

Supplementary Table 1 – Variables obtained from UK Cystic Fibrosis Registry

| **Variable** | **Definition** |
| --- | --- |
| Age | Age at annual review. |
| Gender |  |
| Genotype | Per allele classifications of known alleles. |
| Postcode | Home address post code district. |
| Transplant Evaluated | Evaluated for transplant at annual review. |
| Transplant Received | Recipient of transplant at annual review. |
| *Staphylococcus aureus* status | *S. aureus* as a complication either. Negative, chronic or intermittent. Chronic infection defined as either three or more positive samples in a year. |
| *Pseudomonas aeruginosa* status | *P. aeruginosa* as a complication either. Negative, chronic or intermittent. Chronic infection defined as either three or more positive samples in a year. |
| Allergic bronchopulmonary aspergillosis | Patient considered to have this diagnosis during the last 12 months. |
| Cystic fibrosis related diabetes | Patient considered to have this diagnosis during the last 12 months. |
| Nontuberculous mycobacteria | Detected colonisation at any point during year. |
| Height percentile | As recorded at annual review date. |
| Weight percentile | As recorded at annual review date. |
| BMI percentile | As recorded at annual review date. |
| FVC | Highest value for patient recorded during year. |
| FVC % predicted | Highest value for patient recorded during year. |
| FEV1 | Highest value for patient recorded during year. |
| FEV1 % predicted | Highest value for patient recorded during year. |
| FEF 25-75 | Highest value for patient recorded during year. |
| FEF 25-75 % predicted | Highest value for patient recorded during year. |
| *Burkholderia cepacia* | Detected colonisation at any point during year. |
| *Burkholderia cenocepacia* | Detected colonisation at any point during year. |
| *Burkholderia multivorans* | Detected colonisation at any point during year. |
| NTM species information | Detailed culture information for NTM positive patients. N.b. Only available for 2014 and 2015. |
| NTM treatment details | Detailed treatment information for NTM positive patients. N.b. Only available for 2014 and 2015. |

_Variables obtained from the UK Cystic Fibrosis Registry. NTM species and treatment information was only available for 2014 and 2015. BMI: Body Mass Index; FVC: Forced Vital Capacity; FEV1: Forced Expiratory Volume in 1 second; FEF: Forced Expiratory Flow. NTM: Nontuberculous mycobacteria_

Supplementary Table 2 – Specific strain information 2014 -2015

| **Strain** | **2014 (%)** | **2015 (%)** |
| --- | --- | --- |
| *M. abscessus* + *M. avium* | 3 (2.1) | 1 (0.6) |
| *M. abscessus* + *M. chelonae* | 1 (0.7) | - |
| *M. abscessus* + Unknown | 1 (0.7) | - |
| *M. abscessus* | 72 (51.4) | 55 (35.3) |
| *M. avium* | 16 (11.4) | 13 (8.3) |
| *M. kansasii* | 1 (0.7) | 1 (0.6) |
| Unknown | 46 (32.9) | 86 (55.1) |
| Total | 140 (100) | 156 (100) |

_NTM typing values by year, data visualised in Figure 4._

Supplementary Table 3 – Specific treatment information for 2014 - 2015

| **Year** |  | **All Cases** | **Immuno-supressed** | **IV Induction** | **Amikacin** | **Azithromycin** | **Cefoxitine** | **Clarithromycin** | **Ciprofloxacin** | **Clofazimine** | **Cotrimoxazole** | **Doxycycline** | **Ethambutol** | **Imipenem** | **Isoniazid** | **Levofloxacin** | **Linezolid** | **Meropenem** | **Minocycline** | **Moxifloxacin** | **Rifampicin** | **Tigecycline** |
| --- | --- | --- | --- | --- | --- | --- | --- | --- | --- | --- | --- | --- | --- | --- | --- | --- | --- | --- | --- | --- | --- | --- |
| 2014 | Total | 140 | 4 | 68 | 58 | 30 | 16 | 55 | 32 | 0 | 12 | 4 | 16 | 11 | 2 | 0 | 6 | 26 | 25 | 20 | 15 | 7 |
|  | *Mabsc* | 72 | 2 | 60 | 52 | 24 | 14 | 40 | 30 | 0 | 12 | 4 | 2 | 10 | 0 | 0 | 5 | 26 | 25 | 16 | 3 | 7 |
|  | % | 100 | 2.8 | 83.3 | 72.2 | 33.3 | 19.4 | 55.6 | 41.7 | 0 | 16.7 | 5.6 | 2.8 | 13.9 | 0 | 0 | 6.9 | 36.1 | 34.7 | 22.2 | 4.2 | 9.7 |
|  | *Mac* | 16 | 2 | 6 | 4 | 4 | 2 | 11 | 2 | 0 | 0 | 0 | 10 | 1 | 1 | 0 | 1 | 0 | 0 | 2 | 9 | 1 |
|  | % | 100 | 12.5 | 37.5 | 25.0 | 25.0 | 12.5 | 68.8 | 12.5 | 0 | 0 | 0 | 62.5 | 6.23 | 6.23 | 0 | 6.3 | 0 | 0 | 12.5 | 56.3 | 6.3 |
| 2015 | Total | 156 | 4 | 59 | 49 | 25 | 17 | 36 | 20 | 1 | 14 | 3 | 13 | 7 | 1 | 2 | 3 | 22 | 20 | 18 | 12 | 8 |
|  | *Mabsc* | 55 | 4 | 51 | 46 | 20 | 17 | 28 | 18 | 1 | 14 | 3 | 2 | 7 | 0 | 2 | 3 | 22 | 18 | 17 | 1 | 8 |
|  | % | 100 | 7.3 | 92.7 | 83.6 | 36.4 | 30.9 | 50.9 | 32.7 | 1.8 | 25.5 | 5.5 | 3.6 | 12.7 | 0 | 3.6 | 5.5 | 40.0 | 32.7 | 30.9 | 1.8 | 14.6 |
|  | *Mac* | 13 | 0 | 7 | 3 | 3 | 1 | 8 | 3 | 0 | 0 | 0 | 9 | 0 | 0 | 0 | 0 | 1 | 0 | 0 | 9 | 1 |
|  | % | 100 | 0 | 53.9 | 23.1 | 23.1 | 7.7 | 61.5 | 23.1 | 0 | 0 | 0 | 69.2 | 0 | 0 | 0 | 0 | 7.7 | 0 | 0 | 69.2 | 7.7 |
| Merged | Total | 296 | 8 | 127 | 107 | 55 | 33 | 91 | 52 | 1 | 26 | 7 | 29 | 18 | 3 | 2 | 9 | 48 | 45 | 38 | 27 | 15 |
|  | *Mabsc* | 127 | 6 | 111 | 98 | 44 | 31 | 68 | 48 | 1 | 26 | 7 | 4 | 17 | 0 | 2 | 8 | 48 | 43 | 33 | 4 | 15 |
|  | % | 100 | 4.7 | 87.4 | 77.2 | 34.6 | 24.4 | 53.5 | 37.8 | 0.8 | 20.5 | 5.5 | 3.1 | 13.4 | 0 | 1.6 | 6.3 | 37.8 | 33.9 | 26.0 | 3.1 | 11.8 |
|  | *Mac* | 29 | 2 | 13 | 7 | 7 | 3 | 19 | 5 | 0 | 0 | 0 | 19 | 1 | 1 | 0 | 1 | 1 | 0 | 2 | 18 | 2 |
|  | % | 100 | 6.9 | 44.8 | 24.1 | 24.1 | 10.3 | 65.5 | 17.2 | 0 | 0 | 0.0 | 65.5 | 3.4 | 3.4 | 0 | 3.4 | 3.4 | 0 | 6.9 | 62.1 | 6.9 |

_Treatment information for NTM positive patients from 2014-2015 and merged data._ *_Mabsc_* _-_ *_Mycobacterium abscessus_* _complex_*_, Mac - Mycobacterium avium_* _complex_*_._*
